# Retraction: Treatment of Trigeminal Neuralgia With Stereotactic Radiosurgery Improved Symptoms of Morbihan Syndrome

**DOI:** 10.7759/cureus.r60

**Published:** 2022-07-07

**Authors:** Laura Burgess, Layth Mula-Hussain, Shawn Malone

**Affiliations:** 1 Department of Radiology, Division of Radiation Oncology, University of Ottawa, Ottawa, CAN; 2 Department of Radiology, Division of Radiation Oncology, The Ottawa Hospital Research Institute, Ottawa, CAN; 3 Department of Radiation Oncology, Sultan Qaboos Cancer Centre, Muscat, OMN

This article has been retracted at the request of the authors due to a dispute with the patient featured in this case report. Consent for the case report was obtained from the patient, but the patient felt that there was some misunderstandings regarding their treatment benefits and thus felt the case report was not fully accurate. Upon learning of this from the patient, the authors formally requested that the article be retracted to avoid further misunderstanding. After review, the Cureus editorial office has determined that this request is valid and thus the article has been retracted. We regret any confusion caused by this misunderstanding.

